# Reduction in mRNA Expression of the Neutrophil Chemoattract Factor CXCL1 in *Pseudomonas aeruginosa* Treated Barth Syndrome B Lymphoblasts

**DOI:** 10.3390/biology12050730

**Published:** 2023-05-16

**Authors:** Hana M. Zegallai, Kangmin Duan, Grant M. Hatch

**Affiliations:** 1 Department of Pharmacology & Therapeutics, Children’s Hospital Research Institute of Manitoba, University of Manitoba 753 McDermot Avenue, Winnipeg, MB R3E0T6, Canada; 2 Department of Oral Biology, University of Manitoba, Winnipeg, MB R3E0T6, Canada

**Keywords:** Barth Syndrome, X-linked genetic disease, human B lymphoblasts, *TAFAZZIN*, cardiolipin, chemokine (C-X-C motif) ligand 1, CXCL1, *Pseudomonas aeruginosa*, bacterial activation, immune biology

## Abstract

**Simple Summary:**

Barth Syndrome (BTHS) is a rare X-linked genetic disease in which some patients suffer from severe infections due to neutrophil dysfunction. B cells produce cytokines that attract neutrophils to sites of infection. Here, we examined if B cells from BTHS patients exhibited a reduced ability to express chemokine (C-X-C motif) ligand 1 (CXCL1), a known chemoattractant for neutrophils. We show that B cells from BTHS patients exhibit lowered expression of CXCL1 when stimulated with bacteria compared to control cells. Our findings suggest that an impaired ability of B cells to produce cytokines might contribute to infections in some BTHS patients.

**Abstract:**

Barth Syndrome (BTHS) is a rare X-linked genetic disease caused by a mutation in the *TAFAZZIN* gene, which codes for the protein tafazzin involved in cardiolipin remodeling. Approximately 70% of patients with BTHS exhibit severe infections due to neutropenia. However, neutrophils from BTHS patients have been shown to exhibit normal phagocytosis and killing activity. B lymphocytes play a crucial role in the regulation of the immune system and, when activated, secrete cytokines known to attract neutrophils to sites of infection. We examined the expression of chemokine (C-X-C motif) ligand 1 (CXCL1), a known chemotactic for neutrophils, in Epstein–Barr virus transformed control and BTHS B lymphoblasts. Age-matched control and BTHS B lymphoblasts were incubated with *Pseudomonas aeruginosa* for 24 h and then cell viability, CD27+, CD24+, CD38+, CD138+ and PD1+ surface marker expression and CXCL1 mRNA expression determined. Cell viability was maintained in lymphoblasts incubated in a ratio of 50:1 bacteria:B cells. Surface marker expression was unaltered between control and BTHS B lymphoblasts. In contrast, CXCL1 mRNA expression was reduced approximately 70% (*p* < 0.05) in untreated BTHS B lymphoblasts compared to control and approximately 90% (*p* < 0.05) in bacterial treated BTHS B lymphoblasts compared to the control. Thus, naïve and bacterial-activated BTHS B lymphoblasts exhibit reduced mRNA expression of the neutrophil chemoattractant factor CXCL1. We suggest that impaired bacterial activation of B cells in some BTHS patients could influence neutrophil function via impairing neutrophil recruitment to sites of infection and this could potentially contribute to these infections.

## 1. Introduction

Barth Syndrome (BTHS) is an X-linked genetic disease of young boys [1,2,3,4,5,6,7]. It is caused by mutation in the gene *TAFAZZIN*. The protein product tafazzin is a transacylase enzyme responsible for remodeling the phospholipid cardiolipin [8]. Cardiolipin is a key mitochondrial phospholipid that is essential for the activation of many enzymes of the electron transport chain. As a result, cells from BTHS patients have a reduced ability to produce ATP from oxidative phosphorylation [1,2,3,4,5,6,7]. This leads to the development of cardiomyopathy, skeletal myopathy, growth retardation, 3-methylglucaconic aciduria, and neutropenia.

Although cardiomyopathy is the main pathological problem in BTHS, as most boys develop heart failure, many of these boys (approximately 70%) exhibit severe infection due to neutropenia [9]. Interestingly, neutrophils isolated from BTHS patients have been shown to exhibit normal phagocytosis and killing activity [10]. The neutropenia of BTHS is typically treated with a granulocyte-colony stimulating factor, but in some rare cases infections persist [9]. B lymphocytes are known to influence and modulate neutrophil function through synthesis and secretion of products such as cytokines and chemokines. For example, B lymphoblasts produce chemokine (C-X-C motif) ligand 1 (CXCL1), which is a known chemotactic for neutrophils [11]. In the current study, we examined if BTHS B lymphoblasts exhibited impaired ability to express the neutrophil chemoattractant factor CXCL1. We show that both naïve and *Pseudomonas aeruginosa*-stimulated BTHS B lymphoblasts exhibit impaired expression of CXCL1. We hypothesize that impaired bacterial activation of B lymphocytes could influence the immune function in some BTHS patients and may contribute to infections.

## 2. Materials and Methods

### 2.1. Materials

Epstein–Barr-virus-transformed human control lymphoblasts and Epstein–Barr-virus-transformed BTHS patient lymphoblasts were obtained from the Coriell Institute for Medical Research (Camden, NJ, USA). The cells that were used are outlined in Table 1. Fetal bovine serum (FBS), RPMI 1640 media, antibiotic-antimycotic, and propidium iodide were obtained from Life Technologies Inc. (Burlington, ON, Canada). Anti-CD19-APC, anti-CD24-Pacific blue, anti-CD27-perCP, anti-CD38-APC-CY7, anti-CD138-PE, and anti-PD1-APC antibodies for flow cytometry analysis were purchased from BD Biosciences (Seattle, WA, USA). Unless otherwise indicated, all other reagents used were of analytical grade and were obtained from either ThermoFisher Scientific (Winnipeg, MB, Canada) or Sigma-Aldrich (Oakville, ON, Canada).

### 2.2. Cell Culture and Bacterial Stimulation

The work was performed with approval from the University of Manitoba Environmental Health and Safety Office (Biological Safety Project Approval Certificate #BB0044-2). Cells were grown in RPMI-1640 medium supplemented with 15% FBS and 1% antibiotic-antimycotic at 5% CO_2_ at 37 °C in a Thermo Scientific CO_2_ incubator HEPA Class 200 (Waltham, MA, USA). The medium was replaced every 48 h and cells passaged every five days. Cells were pelleted by centrifugation at 1400 rpm for 10 min at room temperature. Cells were then washed 2X with phosphate buffered saline (PBS) prior to experimental procedures. Control and BTHS lymphoblasts were incubated plus or minus live *Pseudomonas aeruginosa* bacteria (at exponential phase) in ratio 50:1 bacteria:human cells for 24 h. After stimulation, the cells were harvested by centrifugation as above and washed twice with PBS prior to further analysis.

### 2.3. Relative Gene Expression

RNA was isolated from control and BTHS patient lymphoblasts using RNeasy Mini Kit and Qiashredder (Qiagen, Hilden, Germany) homogenizer columns. RT-PCR was performed using the QuantiTect^®^ Probe RT PCR Kit (Qiagen, Hilden, Germany) and the double-stranded DNA stain SYBR green (Qiagen, Hilden, Germany) as indicated by the manufacturer. Relative gene expression analysis was calculated using the 2^−ΔΔCt^ method. The primers that were used for RT-PCR detection as follows: CXCL1: forward, 5′- GAACATCCAAAGTGTGAACGTGAAG-3′; reverse, 5′-TTCAGGAACAGCCACCAGTGAG-3′; β-actin: forward, 5′- GG CGGCACCACCATGTACCCT-3′; reverse, and 5′- AGGGGCCGGACTCGTCA TACT-3′. All primers were obtained from Integrated DNA Technologies (Coralville, IA, USA).

#### Cell Viability and Surface Marker Expression Analysis

Briefly, after 24 h of stimulation with live bacteria, the lymphoblasts were centrifuged at 1400 rpm for 10 min. The pellets were washed with PBS and suspended in 100 µL of PBS. Untreated and treated lymphoblasts were stained with propidium iodide (5 µg/mL) for 5 min in the dark at 4 °C. This was followed by flow cytometry analysis (see below). Surface marker expression was measured in untreated and treated lymphoblasts by staining the cell surface with anti-CD19-APC, anti-CD24-Pacific blue, anti-CD27-perCP, anti-CD38-APC-CY7, anti-CD138-PE, and anti-PD1-APC as per the manufacturer’s instructions. Cells were then analyzed by flow cytometry at the Flow Cytometry Core Facility in the Rady Faculty of Health Sciences at the University of Manitoba, using a BD FACS Canto II instrument. FlowJo software was used for data analysis.

### 2.4. Statistical Analysis

All experimental results were expressed as mean ± SD. A two-tailed unpaired Student’s t test was performed to compare between groups. The level of significance was defined as *p* < 0.05.

## 3. Results

**Figure 1:**
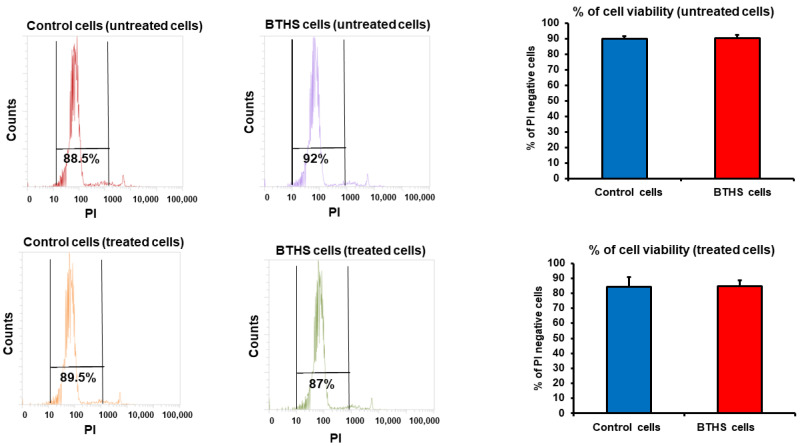
Cell viability of control and BTHS lymphoblasts incubated with bacteria. Control and BTHS lymphoblasts were incubated for 24 h plus or minus *Pseudomonas aeruginosa* and cell viability determined using flow cytometry analysis as described in Materials and Methods. Data represent the mean ± SD, *n* = 3.

Initially, we examined the cell viability of the control and BTHS lymphoblasts treated with *Pseudomonas aeruginosa*. The control and BTHS lymphoblasts were incubated for 24 h in the absence or presence of a 50:1 ratio of *Pseudomonas aeruginosa*:B cells and then stained with propidium iodide and viability determined using flow cytometry analysis. Cell viability was maintained in both the control and BTHS B lymphoblasts incubated for 24 h with *Pseudomonas aeruginosa* (Figure 1).

**Figure 2:**
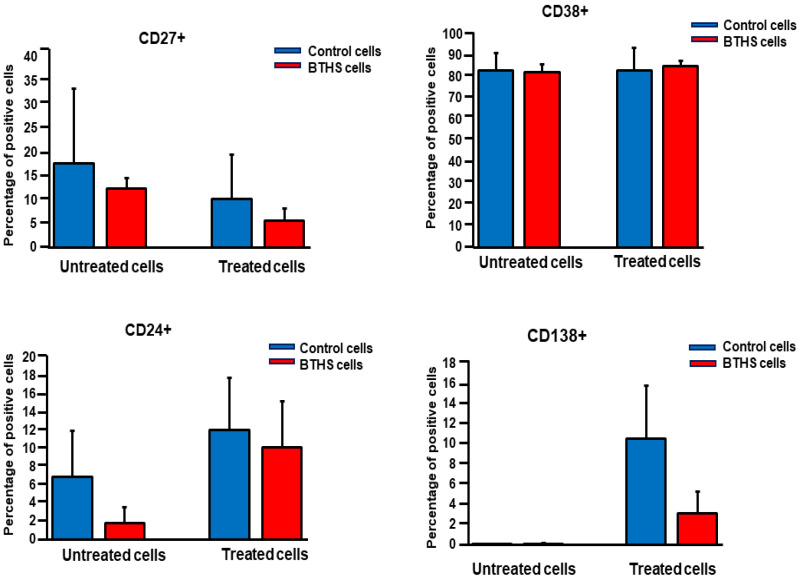

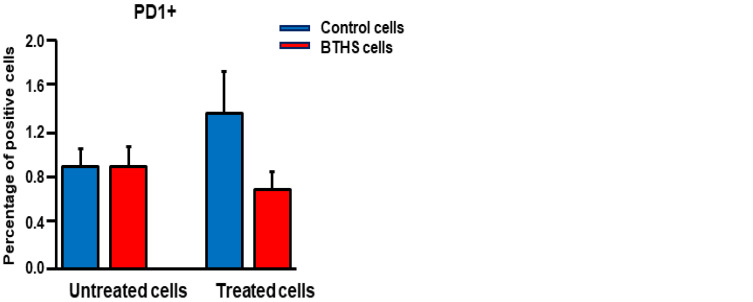
Surface marker expression in control and BTHS lymphoblasts incubated with bacteria. Control and BTHS lymphoblasts were incubated for 24 h plus or minus *Pseudomonas aeruginosa* and surface marker expression of CD27+, CD24+, CD38+, CD138+ and PD1+ determined using flow cytometry analysis as described in Materials and Methods. Data represent the mean ± SD, *n* = 3.

Activation of B lymphocytes with bacteria results in the expression of surface markers such as CD138+. We next examined expression of surface markers on control and BTHS lymphoblasts treated with *Pseudomonas aeruginosa*. Control and BTHS lymphoblasts were incubated for 24 h with *Pseudomonas aeruginosa* and surface expression of CD27+, CD24+, CD38+, CD138+ and PD1+ determined using flow cytometry analysis. Surface expression of CD27+, CD24+, CD38+ and PD1+ was unaltered between untreated control and BTHS lymphoblasts (Figure 2 and Figure S1). In addition, when cells we treated with *Pseudomonas aeruginosa*, the surface expression of CD27+, CD24+, CD38+ and PD1+ was unaltered between the control and BTHS lymphoblasts. As expected, CD138+ was minimally expressed in the unstimulated control and BTHS cells. When cells were treated with *Pseudomonas aeruginosa*, the surface expression of CD138+ was upregulated in both the control and BTHS lymphoblasts.

**Figure 3:**
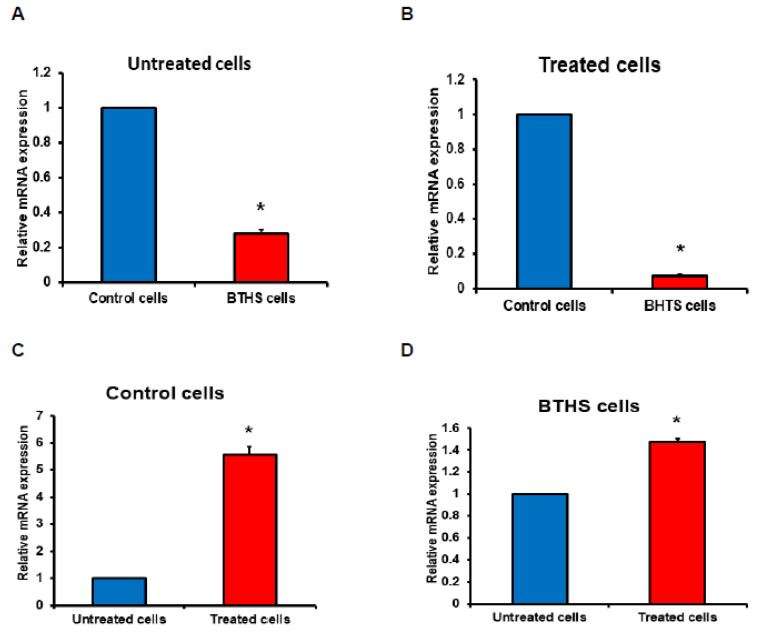
CXCL1 expression is reduced in bacterial-treated BTHS patient lymphoblasts. Control and BTHS lymphoblasts were incubated for 24 h plus or minus *Pseudomonas aeruginosa* and CXCL1 mRNA expression determined as described in Materials and Methods. Relative CXCL1 mRNA expression in untreated cells (**A**). Relative CXCL1 mRNA expression in treated cells (**B**). Relative CXCL1 mRNA expression in *Pseudomonas aeruginosa* treated control cells compared to untreated control cells (**C**). Relative CXCL1 mRNA expression in *Pseudomonas aeruginosa* treated BTHS cells compared to untreated BTHS cells (**D**). Data represent the mean ±SD, *n* = 3. * *p* < 0.05.

Next, we examined the mRNA expression of CXCL1 in control and BTHS lymphoblasts treated with *Pseudomonas aeruginosa*. The relative basal mRNA expression of CXCL1 in untreated naïve BTHS lymphoblasts was 70% lower compared to untreated naïve control lymphoblasts (Figure 3A). In addition, the relative mRNA expression of CXCL1 in *Pseudomonas aeruginosa*-treated BTHS lymphoblasts was 90% lower compared to *Pseudomonas aeruginosa*-treated control lymphoblasts (Figure 3B). When control cells were compared, relative CXCL1 mRNA expression was elevated 7-fold by *Pseudomonas aeruginosa* treatment of control cells compared to untreated control cells (Figure 3C). When BTHS cells were compared, the relative CXCL1 mRNA expression was elevated by only 40% in *Pseudomonas aeruginosa*-treated BTHS cells compared to untreated BTHS cells (Figure 3D), indicating a reduced ability of BTHS B cells to express CXCL1 mRNA compared to the control cells. Thus, naïve and *Pseudomonas aeruginosa*-stimulated BTHS B lymphoblasts exhibit impaired mRNA expression of CXCL1.

## 4. Discussion

Tafazzin deficiency is known to negatively impact the function of a broad range of immune cells including mesenchymal stem cells, mast cells, CD8+ T cells and B cells [12,13,14,15]. Crosstalk between neutrophils and B cells occurs in several diseases including rheumatoid arthritis and antineutrophil cytoplasmic antibody-associated vasculitis [16,17]. CXCL1 is a CXC family member that acts as a key chemoattractant for immune cells, especially neutrophils and other non-hematopoietic cells to sites of injury or infection and plays a key role in the regulation of immune and inflammatory responses [18,19]. A previous study had indicated that neutrophils isolated from BTHS patients exhibited normal phagocytosis and killing activity [10]. Moreover, a study examining the clinical data, routine blood counts, and responses to granulocyte-colony stimulating factor therapy from 88 affected BTHS boys concluded that susceptibility to infections was due only in part to neutropenia since in some instances infection occurred despite consistent prevention of neutropenia by granulocyte-colony-stimulating factor therapy [9]. A more recent study examined myeloid progenitor development within the fetal liver of TAFAZZIN knockout animals as well as within the adult bone marrow of wildtype recipients transplanted with TAFAZZIN knockout hematopoietic stem cells [20]. Although TAFAZZIN knockout neutrophils demonstrated the expected differences in CL maturation, no significant differences in neutrophil development or neutrophil function, including the production of cytokines, was observed. However, transcriptomic analysis of the TAFAZZIN-deficient neutrophil progenitors demonstrated an upregulation of markers of endoplasmic reticulum (ER) stress and increased sensitivity to certain ER stress-mediated and non-ER stress-mediated triggers of apoptosis. Interestingly, elevated ER stress is associated with immunosuppression [21].

In the current study, we observed mRNA expression of CXCL1 in both naïve and *Pseudomonas aeruginosa*-activated control and BTHS lymphoblasts. However, the degree of CXCL1 mRNA expression was markedly lower in naïve or bacterial-activated BTHS lymphoblasts compared to naïve or bacterial-activated control lymphoblasts, indicating an impaired ability to produce CXCL1 by BTHS patient B lymphoblasts.

PD1+ surface marker expression is associated with down-regulation of the immune system [22]. For example, PD1+ expression on activated B lymphocytes was shown to inhibit CD4+ and CD8+ T cell proliferation [23]. The degree of expression of PD1+ appeared lower in *Pseudomonas aeruginosa*-incubated BTHS cells than control cells, although this was not statistically significant. The presence of CD138+ expression on B lymphocytes is indicative of an active antibody-secreting phenotype of B cells. Interestingly, although *Pseudomonas aeruginosa* treatment resulted in the expression of CD138+ in both control and BTHS cells, the degree of expression appeared lower in BTHS cells than control cells, although this was not statistically significant. These results are consistent with the recent identification of a BTHS patient with persistent B cell lymphopaenia and hypogammaglobulinaemia [24]. The patient experienced repeated bacterial and viral infections and required subcutaneous immunoglobulin replacement for ongoing hypogammaglobulinaemia. Thus, it is possible that reduced CD138+ expression on B lymphocytes might be linked to attenuated antibody production in some BTHS patients.

A limitation of our study is the use of Epstein–Barr virus transformed control and BTHS lymphoblasts as opposed to primary human BTHS B lymphocytes. Epstein–Barr virus transformation of primary B lymphocytes results in differential expression of several genes compared to that of normal human B lymphocytes [11]. These include 22 up-regulated genes and 16 down-regulated genes (one of which is CXCL1), which control processes such as the cell cycle, mitosis, the cytokine–cytokine pathway and the immunity response, thus hindering host immune function and secretion of cytokines. We recently observed that Epstein–Barr virus transformation of control and BTHS human B lymphocytes exhibited impaired responsiveness to lipopolysacharride-mediated or CpG-DNA-mediated surface marker expression [25]. Thus, the responsiveness of the control and BTHS Epstein–Barr virus transformed lymphoblasts to *Pseudomonas aeruginosa* bacterial activation may additionally be impaired compared to non-transformed naïve B lymphocytes.

## 5. Conclusions

We have observed reduced CXCL1 mRNA expression, a known chemoattractant of neutrophils, in naïve and in *Pseudomonas aeruginosa*-activated B lymphoblasts from BTHS patients. We hypothesize that a reduced expression of CXCL1 by B lymphocytes may, in part, help to explain why BTHS boys suffer from infections even when neutrophil counts are at or near normal levels.

## Figures and Tables

**Table 1:** Patient cell lines used in this study.

Cell Line Identifier	Phenotype	TAFAZZIN Mutation	Age at Harvest
07535	Healthy control	None	15 years
07491	Healthy control	None	17 years
16408	Healthy control	None	12 years
22192	BTHS	Exon 3, Substitution of cystine for arginine	10 years
22193	BTHS	Exon 6, premature stop codon	10 years
22194	BTHS	Complete deletion of TAFAZZIN gene	9 years

## Data Availability

All data and supporting data are reported in the manuscript.

## Supplementary Materials

The following supporting information can be downloaded at: https://www.mdpi.com/article/10.3390/biology12050730/s1, Figure S1: Representative flow cytometry images of surface markers expressed in untreated and *Pseudomonas aeruginosa* treated control and BTHS lymphoblasts.

## References

[1] Barth P.G., Scholte H.R., Berden J.A., Van der Klei-Van Moorsel J.M., Luyt-Houwen I.E.M., Veer-Korthof E.T.V.T., Van der Harten J.J., Sobotka-Plojhar M.A. (1983). An X-linked mitochondrial disease affecting cardiac muscle, skeletal muscle and neutrophil leucocytes. J. Neurol. Sci..

[2] Kelley R.I., Cheatham J.P., Clark B.J., Nigro M.A., Powell B.R., Sherwood G.W., Sladky J.T., Swisher W.P. (1991). X-linked dilated cardiomyopathy with neutropenia, growth retardation, and 3-methylglutaconic aciduria. J. Pediatr..

[3] Clarke S.L., Bowron A., Gonzalez I.L., Groves S.J., Newbury-Ecob R., Clayton N., Martin R.P., Tsai-Goodman B., Garratt V., Ashworth M. (2013). Barth Syndrome. Orphanet J. Rare Dis..

[4] Hauff K.D., Hatch G.M. (2006). Cardiolipin metabolism and Barth Syndrome. Prog. Lipid Res..

[5] Zegallai H.M., Hatch G.M. (2021). Barth syndrome: Cardiolipin, cellular pathophysiology, management, and novel therapeutic targets. Mol. Cell. Biochem..

[6] Taylor C., Rao E.S., Pierre G., Chronopoulou E., Hornby B., Heyman A., Vernon H.J. (2022). Clinical presentation and natural history of Barth Syndrome: An overview. J. Inherit. Metab. Dis..

[7] Thompson R., Jefferies J., Wang S., Pu W.T., Takemoto C., Hornby B., Heyman A., Chin M.T., Vernon H.J. (2022). Current and future treatment approaches for Barth syndrome. J. Inherit. Metab. Dis..

[8] Xu Y., Malhotra A., Ren M., Schlame M. (2006). The enzymatic function of tafazzin. J. Biol. Chem..

[9] Steward C.G., Groves S.J., Taylor C.T., Maisenbacher M.K., Versluys B., Newbury-Ecob R.A., Ozsahin H., Damin M.K., Bowen V.M., McCurdy K.R. (2019). Neutropenia in Barth syndrome: Characteristics, risks, and management. Curr. Opin. Hematol..

[10] Kuijpers T.W., Maianski N.A., Tool A.T., Becker K., Plecko B., Valianpour F., Wanders R.J., Pereira R., Van Hove J., Verhoeven A.J. (2000). Neutrophils in Barth syndrome (BTHS) avidly bind annexin-V in the absence of apoptosis. Blood.

[11] Dai Y., Tang Y., He F., Zhang Y., Cheng A., Gan R., Wu Y. (2012). Screening and functional analysis of differentially expressed genes in EBV-transformed lymphoblasts. Virol. J..

[12] Zegallai H.M., Abu-El-Rub E., Olayinka-Adefemi F., Cole L.K., Sparagna G.C., Marshall A.J., Hatch G.M. (2022). Tafazzin deficiency in mouse mesenchymal stem cells promote reprogramming of activated B lymphocytes toward immunosuppressive phenotypes. FASEB J..

[13] Zegallai H.M., Abu-El-Rub E., Cole L.K., Field J., Mejia E.M., Gordon J.W., Marshall A.J., Hatch G.M. (2021). Tafazzin deficiency impairs mitochondrial metabolism and function of lipopolysaccharide activated B lymphocytes in mice. FASEB J..

[14] Corrado M., Edwards-Hicks J., Villa M., Flachsmann L.J., Sanin D.E., Jacobs M., Baixauli F., Stanczak M., Anderson E., Azuma M. (2020). Dynamic cardiolipin synthesis is required for CD8(+) T Cell immunity. Cell Metab..

[15] Maguire A.R.R., Crozier R.W.E., Hunter K.D., Claypool S.M., Fajardo V.A., LeBlanc P.J., MacNeil A.J. (2021). Tafazzin modulates allergen-induced mast cell inflammatory mediator secretion. ImmunoHorizons.

[16] Karmakar U., Vermeren S. (2021). Crosstalk between B cells and neutrophils in rheumatoid arthritis. Immunology.

[17] Witko-Sarsat V., Daniel S., Noël L.-H., Mouthon L. (2009). Neutrophils and B lymphocytes in ANCA-associated vasculitis. Apmis.

[18] Moser B., Clark-Lewis I., Zwahlen R., Baggiolini M. (1990). Neutrophil-activating properties of the melanoma growth-stimulatory activity. J. Exp. Med..

[19] Schumacher C., Clark-Lewis I., Baggiolini M., Moser B. (1992). High- and low-affinity binding of GRO alpha and neutrophil-activating peptide 2 to interleukin 8 receptors on human neutrophils. Proc. Natl. Acad. Sci. USA.

[20] Sohn J., Milosevic J., Brouse T., Aziz N., Elkhoury J., Wang S., Hauschild A., Van Gastel N., Cetinbas M., Tufa S.F. (2022). A new murine model of Barth syndrome neutropenia links TAFAZZIN deficiency to increased ER stress-induced apoptosis. Blood Adv..

[21] Lee B.-R., Chang S.-Y., Hong E.-H., Kwon B.-E., Kim H.M., Kim Y.-J., Lee J., Cho H.-J., Cheon J.-H., Ko H.-J. (2014). Elevated endoplasmic reticulum stress reinforced immunosuppression in the tumor microenvironment via myeloid-derived suppressor cells. Oncotarget.

[22] Trivedi M.S., Hoffner B., Winkelmann J.L., Abbott M.E., Hamid O., Carvajal R.D. (2015). Programmed death 1 immune checkpoint inhibitors. Clin. Adv. Hematol. Oncol..

[23] Wang X., Wang G., Wang Z., Liu B., Han N., Li J., Lu C., Liu X., Zhang Q., Yang Q. (2019). PD-1-expressing B cells suppress CD4+ and CD8+ T cells via PD-1/PD-L1-dependent pathway. Mol. Immunol..

[24] Kudlaty E., Agnihotri N., Khojah A. (2022). Hypogammaglobulinaemia and B cell lymphopaenia in Barth syndrome. BMJ Case Rep..

[25] Zegallai H.M., Hatch G.M. (2022). Impaired surface marker expression in stimulated Epstein-Barr virus transformed lymphoblasts from Barth Syndrome patients. Sci. Rep..

