# Supplementary material for: Reduction in mRNA Expression of the Neutrophil Chemoattract Factor CXCL1 in Pseudomonas aeruginosa Treated Barth Syndrome B Lymphoblasts
Source: Biology (Basel). 2023 May 16;12(5):730. doi: 10.3390/biology12050730 (PMC10215251; doi:10.3390/biology12050730)

Supplementary Figure S1. Representative flow cytometry images of surface markers expressed in untreated and *Pseudomonas aeruginosa* treated control and BTHS lymphoblasts.

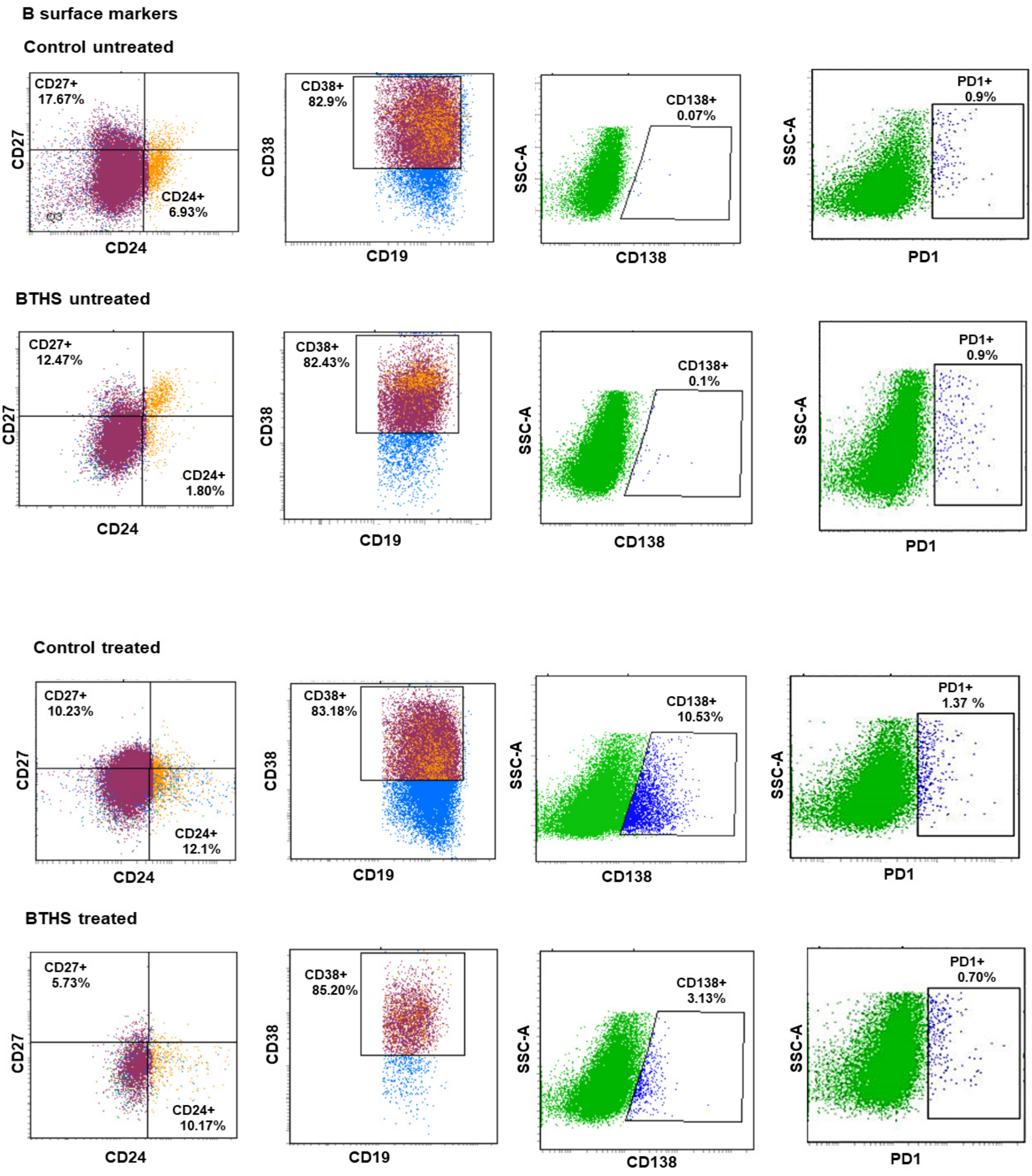

Supplement: Supplementary file 1 [file biology-12-00730-s001.zip › biology-2354690-supplementary.pdf]
